# Reproducibility of Retinol Binding Protein 4 and Omentin-1 Measurements over a Four Months Period: A Reliability Study in a Cohort of 207 Apparently Healthy Participants

**DOI:** 10.1371/journal.pone.0138480

**Published:** 2015-09-24

**Authors:** Clemens Wittenbecher, Romina di Giuseppe, Ronald Biemann, Juliane Menzel, Maria Arregui, Juliane Hoffmann, Krasimira Aleksandrova, Heiner Boeing, Berend Isermann, Matthias B. Schulze, Cornelia Weikert

**Affiliations:** 1 Department of Molecular Epidemiology, German Institute of Human Nutrition Potsdam-Rehbruecke, Nuthetal, Germany; 2 German Center for Diabetes Research (DZD), Neuherberg, Germany; 3 Research Group Cardiovascular Epidemiology, German Institute of Human Nutrition Potsdam-Rehbruecke, Nuthetal, Germany; 4 Department for Clinical Chemistry and Pathobiochemistry, Otto-von-Guericke-University Magdeburg, Magdeburg, Germany; 5 Department of Epidemiology, German Institute of Human Nutrition Potsdam-Rehbruecke, Nuthetal, Germany; 6 Institute of Social Medicine, Epidemiology, and Health Economics, Charité University Medical Center, Berlin, Germany; Johns Hopkins University School of Medicine, UNITED STATES

## Abstract

The reliability of single time point measurements of the novel adipokines retinol-binding protein 4 and omentin-1 in the blood has not been evaluated in large samples yet. The present study aimed to assess the amount of biological variation of these two adipokines within individuals. The study sample comprised 207 participants (124 women and 83 men) from Potsdam (Germany) and surrounding areas, with an average age of 56.5 years (SD 4.2). Blood samples were collected from each participant twice, approximately four months apart. Using enzyme linked immunosorbent assays, the concentrations of retinol-binding protein 4 and omentin-1 were determined in EDTA plasma. As indicators of reliability, intraclass correlation coefficients (ICCs) were calculated from the repeated biomarker measurements. The ICCs for repeated retinol-binding protein 4 and omentin-1 measurements were 0.77 (95% CI 0.71, 0.82) and 0.83 (95% CI 0.78, 0.87), respectively, indicating for both adipokines excellent reliability. ICCs were stable across strata according to sex, age, BMI, and blood pressure. Thus, for epidemiological studies it seems reasonable to rely on concentrations of retinol-binding protein 4 and omentin-1 in samples from a single time point if repeated measurements are not available.

## Introduction

Over the last decade an endocrine function of the adipose tissue has emerged. Among adipose tissue-derived signaling factors, the novel adipokines omentin-1 and retinol-binding protein 4 (RBP4) are of special interest for their possible role in systemic metabolic regulation. Omentin-1 is a 35 kDa glycoprotein [1] which is mainly expressed in visceral adipose tissue [2]. Inverse associations of omentin-1 with body fatness, metabolic syndrome, type 2 diabetes and CVD were reported from cross-sectional human studies [3–6]. RBP4 is a 21 kDa protein with the highest expression rate in the liver, and the second highest rate in adipose tissue [7]. Its main function is the extracellular transport of retinol. More recently, RBP4 was further characterized as novel adipokine [8] which was directly associated with components of the metabolic syndrome [9–12]. Furthermore, RBP4 levels were also elevated in patients with chronic kidney diseases [13], raising the question to which extent the direct association of RBP4 levels with prevalent type 2 diabetes might be explainable by early renal complications [14]. Still, prospective studies have reported longitudinal associations of RBP4 with the incidence of type 2 diabetes [15, 16] and cardiovascular disease (CVD) [17] in humans. Thus, associations of omentin-1 and RBP4 with cardio-metabolic diseases are suggested but incompletely understood and active research regarding these two adipokines can be awaited.

In observational studies, however, the assessment of biomarker concentrations often relies on blood samples from a single time point. Hence, the interpretability of such measurements with regard to biological differences between study participants depends on the variability of the biomarker concentrations within individuals. Intraindividual variation might depend on environmental factors such as season, daytime, and dietary exposures or endogenous factors such as health status, menstrual cycle, and stress level among others, which are typically not controlled in observational studies. Therefore, the STROBE-ME guidelines explicitly recommended reporting on the long term variability of repeated measurements within persons for the biomarkers applied in epidemiological studies [18]. The biological variation of circulating concentrations of RBP4 and omentin-1 within individuals has not been systematically investigated in large samples yet. Hence, our study aimed to evaluate the within person reproducibility of measurements of circulating RBP4 and omentin-1. This aim was achieved by analyzing measurements of these two adipokines in two blood samples per individual taken several months apart in a group of 207 middle-aged white participants.

## Methods

### Study population

The study population (n = 207, including 124 women and 83 men) consisted of a random sample of apparently healthy EPIC-Potsdam participants who were invited to take part in a validation study in 2007. All participants were under 64 years of age, had no history of cardiovascular events, no impaired mobility, and had systolic and diastolic blood pressure below 180 mmHg and 110 mmHg, respectively. Informed consent was obtained from all participants, and the study was approved by the ethics committee of the Medical Society of the State of Brandenburg (Germany). Recruitment and sampling procedures have been described elsewhere [19]. Briefly, the examinations included anthropometric measurements, blood pressure measurement, and repeated sampling of venous blood approximately four months apart (median 119 days; 5^th^, 95^th^ percentile: 98, 153 days). The first blood samples were taken between October 2007 and March 2008 and the second samples between February and July 2008. Approximately 90% of the participants were fasted for at least 8h at time of blood sampling.

### Biomarker measurements

Plasma samples were collected on EDTA and stored at -80°C by qualified medical personnel. Plasma concentrations of adipokines were measured in commercially available enzyme linked immunosorbent assays (ELISA) at the Institute of Clinical Chemistry, University Magdeburg, according to the manufacturer’s instructions. For RBP4 a competitive ELISA by Biovendor (Brno, Czech Republic) was used with intra-assay coefficients of variation (CV) between 2.6% and 9.2% (5 samples, 4 replicates), inter-assay CVs between 3.5% and 10.3% (5 samples, 4 separate assays) and a lower limit of detection (LOD) of 1 ng/ mL according to the manufacturer. For omentin-1 a sandwich ELISA by Biovendor (Brno, Czech Republic) was used with intra-assay CVs between 3.2% and 4.1% (8 samples, two replicates), inter-assay CVs between 4.4% and 4.8% (8 samples, 2 separate assays) and a LOD of 0.5 ng/mL according to the manufacturer.

### Statistical analysis

All analyses were conducted with SAS version 9.4 (Statistical Analysis System; SAS Institute Inc.).

A random effects ANOVA with adipokine-concentration as dependent variable and study participant as explanatory factor was used to estimate the variance components explained by within person and between person differences, respectively. The CV was calculated as root of the mean square error of residuals from the aforementioned ANOVA and expressed as percentage [20]. The intraclass correlation coefficient (ICC) was calculated by subtracting within person variance from between person variance and dividing the result by the total variance. In agreement with the literature, we considered CVs below 20% as desirable and ICCs above 0.75 to indicate excellent reliability [20]. The reliability parameters of repeated measurements of RBP4 and omentin-1 were also evaluated across strata according to sex, age, BMI, blood pressure, and time-interval between measurement time points to provide general information on the robustness of the results. In sensitivity analyses, we also excluded the 5% most extreme observations (2.5% highest and 2.5% lowest values) of all RBP4 and omentin-1 measurements, respectively, as well as non-fasting participants. Furthermore, the differences between repeated measurements within a person were plotted against the individual means with the range of agreement defined as mean ± 1.96 SD as proposed by Bland and Altman.

In order to demonstrate the usage of ICCs to quantitatively estimate the bias in observed relative risks (RR) that is due to biological variability of the marker we applied the following formula [21]:

$$RR_{true}=e^{\left(lnRR_{observed}*\frac{1}{ICC}\right)}$$

## Results

From the initial sample of 207 participants one individual had missing values for plasma omentin-1 concentrations and was therefore excluded from all subsequent analyses leaving an analytical sample of 206 participants. Table 1 summarizes basic characteristics of the study population including adipokine measurements. Briefly, the average age of participants was 55 years (SD 4.2), 59% were women, 60% were overweight or obese, and 92% had not eaten for at least 8 hours at the time of blood draw. Men were older and had a higher BMI than women. The average concentrations of RBP4 were 50.8 μg/mL (95% CI 48.8, 52.9) in samples from the first blood draw and 50.0 μg/mL (95% CI 48.0, 52.0) in samples from the second blood draw (*p* for difference 0.21); the average concentrations of omentin-1 were 401.0 ng/mL (95% CI 383.6, 418.4) in samples from the first blood draw and 396.8 ng/mL (95% CI 379.4, 414.2) in samples from the second blood draw (*p* for difference 0.41), respectively. At both time points the average RBP4 concentrations were higher while the average omentin-1 concentrations were lower among men compared to women (Table 1).

**Table 1:** Baseline characteristics of the study population.

	All (n = 206)		Men (n = 83)		Women (n = 123)	
RBP4^a^ at t1^b^ [μg/mL]	50.8	(48.8, 52.9)^c^	53.1	(49.7, 56.5)	49.3	(46.8, 51.9)
RBP4 at t2 ^d^ [μg/mL]	50.0	(48.0, 52.0)	53.4	(50.1, 56.6)	47.7	(45.3, 50.1)
Omentin-1 at t1 [ng/mL]	401.0	(383.6, 418.4)	384. 6	(358.1, 411.0)	412.2	(389.0, 435.3)
Omentin-1 at t2 [ng/mL]	396.8	(379.4, 414.2)	396.5	(368.4, 424.6)	397.0	(374.6, 419.4)
Interval (t1-t2) [days]	119	(98–152)^e^	119	(103–154)	119	(98–147)
Age [years]	56.5	(55.9, 57.0)	58.0	(57.4, 58.7)	55.4	(54.6, 56.2)
BMI^f^ [kg/m^2^]	26.5	(25.9, 27.0)	27.6	(26.8, 28.3)	25.8	(25.0, 26.5)
Waist circumference	93.0	(91.2, 94.7)	101.7	(99.4, 103.9)	87.1	(85.1, 89.0)
Systolic BP^g^ [mmHg]	135.3	(133.4, 137.3)	138.9	(135.7, 142.1)	132.9	(130.5, 135.3)
Diastolic BP [mmHg]	87.6	(86.3, 88.9)	90.2	(87.9, 92.4)	85.9	(84.3, 87.4)
sports (summer) [h/w]^h^	1.8	(1.5, 2.2)	1.7	(1.1, 2.3)	1.9	(1.4, 2.3)
sports (winter) [h/w]	1.7	(1.4, 2.0)	1.6	(1.1, 2.1)	1.8	(1.3, 2.2)
Non-fasted	10%		13%		8%	

^a^RBP4: retinol-binding protein 4.

^b^t1: time point 1 (first occasion of blood-sampling).

^c^Mean (95% CI), all such values.

^d^t2: time point 2 (second occasion of blood-sampling).

^e^Median (5^th^-95^th^ Percentile), all such values.

^f^BMI: body mass index.

^g^BP: blood pressure.

^h^[h/w]: hours per week.

**Fig 1:**
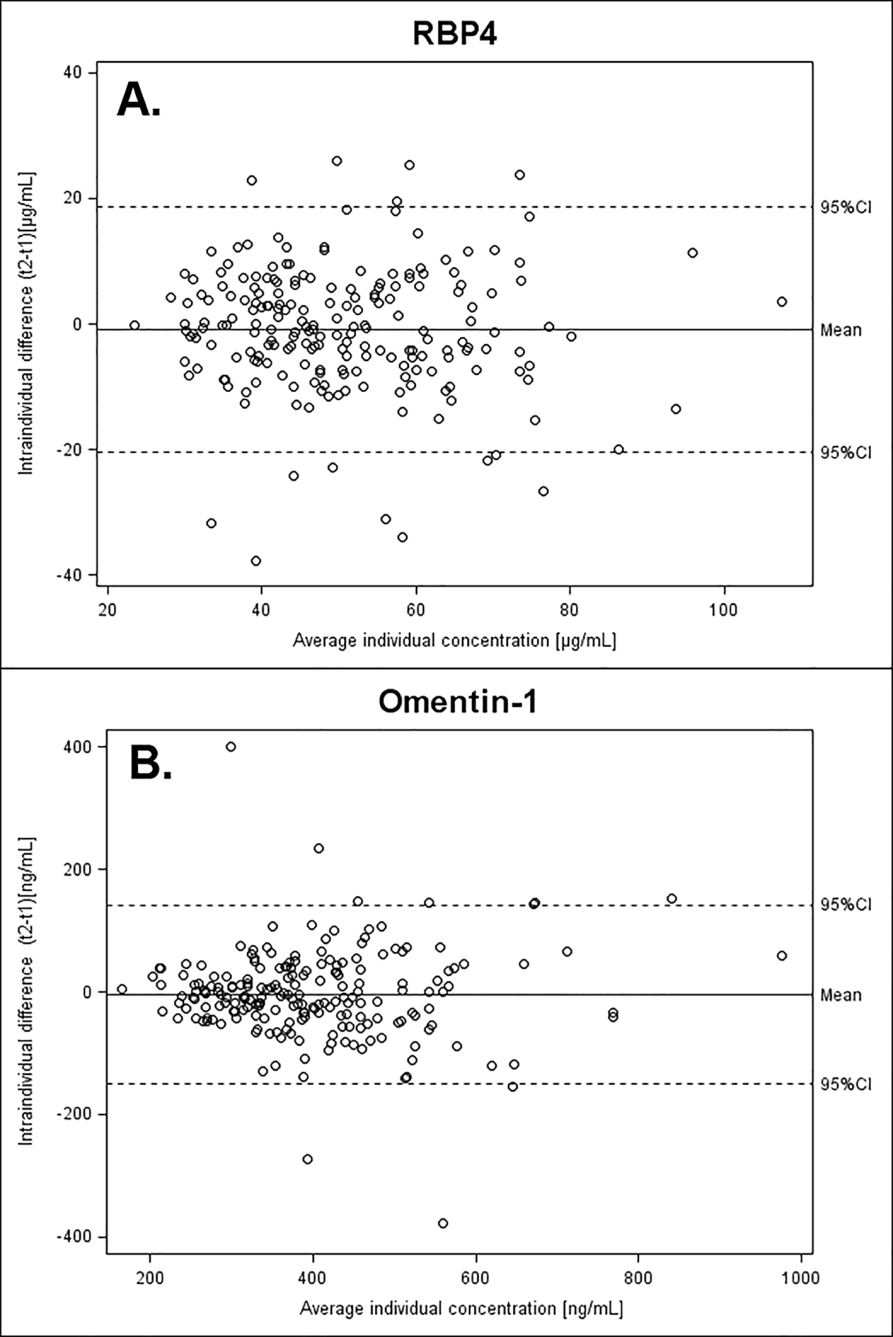
Bland-Altman-Plot. Agreement of repeated biomarker measurements in individuals in relation to the overall variability of measurements; agreement was calculated as difference in plasma-concentrations between the two blood-sampling occasions (t2-t1) within individuals; range of agreement was defined as mean ± 1.96 SD and is marked by the dashed lines. **A**: retinol-binding protein 4 (RBP4); **B**: omentin-1.

The agreement between repeated adipokine measurements within individuals in relation to individual means was visualized in Bland-Altman-Plots (Fig 1). The Bland-Altman-Plots do not show clustering of differences at certain ranges of the adipokine concentrations.

Table 2 shows reliability parameters of the repeated adipokine measurements. The ICCs for repeated RBP4 and omentin-1 measurements were 0.77 (95% CI 0.71, 0.82) and 0.83 (95% CI 0.78, 0.87), respectively. The CVs for intraindividual measurement variation between repeated measurements were 14% for RBP4 and 13% for omentin-1. These results were robust across strata according to sex, age, BMI, blood pressure (diastolic and systolic, respectively), and time-interval between measurement time points (Table 2); i.e. the ICCs within strata were all within the 95% CI of the full cohort ICC. Neither excluding the 5% most extreme observations (2.5% highest and 2.5% lowest values) of all RBP4 and omentin-1 measurements, respectively, nor excluding non-fasted participants from the analyses substantially affected the results.

**Table 2:** Reliability parameters of repeated measurements of RBP4 and omentin-1.

		RBP4		Omentin-1	
Strata	n	CV_RBP4_ ^a^	ICC_RBP4_ (95% CI)	CV_Omentin-1_	ICC_Omentin-1_ (95% CI)
All	206	14%	0.77 (0.71, 0.82)	13%	0.83 (0.78, 0.87)
Gender					
Men	83	13%	0.80 (0.71, 0.87)	13%	0.84 (0.76, 0.89)
Women	123	15%	0.74 (0.65, 0.81)	13%	0.83 (0.77, 0.88)
BMI^b^ [kg/cm^2^]					
≤ 25	80	13%	0.85 (0.78, 0.90)	12%	0.83 (0.75, 0.89)
> 25	126	15%	0.71 (0.61, 0.79)	14%	0.83 (0.77, 0.88)
Age					
≤56.46 (mean)	94	15%	0.77 (0.67, 0.84)	12%	0.86 (0.80, 0.90)
> 56.46 (mean)	112	13%	0.77 (0.68, 0.84)	14%	0.81 (0.74, 0.86)
Diastolic BP^c^ [mmHg]					
≤ 87.57 (mean)	94	16%	0.72 (0.61, 0.80)	15%	0.80 (0.71, 0.86)
> 87.57 (mean)	112	13%	0.82 (0.75, 0.87)	11%	0.87 (0.82, 0.91)
Systolic BP [mmHg]					
≤ 135.31 (mean)	101	13%	0.80 (0.72, 0.86)	14%	0.81 (0.73, 0.87)
> 135.31 (mean)	105	15%	0.75 (0.65, 0.82)	12%	0.85 (0.79, 0.90)
Time-interval [days]					
< 119 (median)	99	13%	0.81 (0.73, 0.87)	14%	0.76 (0.66, 0.83)
≥ 119 (median)	107	15%	0.71 (0.60, 0.79)	12%	0.89 (0.84, 0.92)

Coefficient of variation (CV) and intraclass correlation coefficient (ICC) of repeated biomarker measurements

^a^RBP4: retinol-binding protein 4.

^b^BMI: body mass index.

^c^BP: blood pressure.

We also calculated ICC-corrected (“true”) RRs from hypothetically observed RRs of 1.5, 2.5, and 3.5, respectively. Correcting these observed RRs for the RBP4-related ICC of 0.77 resulted in RRs of 1.69, 3.29, and 5.09, respectively, corresponding to a change in estimate of 12.7%, 31.6%, and 45.4%. Correcting the hypothetically observed RRs for the omentin-1-related ICC of 0.80 resulted in “true” RRs of 1.66, 3.14, and 4.79, respectively, corresponding to a change in estimate of 10.7%, 25.6%, and 36.4%.

## Discussion

In our study in a middle aged white population, measurements of circulating concentrations of RBP4 and omentin-1 were highly reproducible in individuals over a period of several months, indicated by low CV and excellent ICC for both signaling factors. These results were robust across strata according to age, sex, BMI, blood pressure, and time-interval between repeated blood-sampling. Thus, the study is one of the first that provided detailed data on the reliability of measurements of RBP4 and omentin-1 applicable in observational study settings with a single blood draw.

In our study, the CVs for repeated measurements of RBP4 and omentin-1 several months apart were consistently below 20% and ICCs were over 0.75 corresponding to an excellent reliability [20]. Our results indicate that the biological variance of RBP4 and omentin-1 within individuals is sufficiently low compared to the difference of the circulating concentrations between individuals.

Associations of RBP4 and omentin-1 with age, sex, BMI, and systolic as well as diastolic blood pressure were reported [6, 22–25]. An important question is if the reliability might depend on these factors. The stability of reliability-related parameters (ICC and CV) across strata according to these factors in our study did not indicate such dependencies. Furthermore, our sensitivity analysis over strata according to time-interval between the two blood-sampling occasions did not indicate that reliability substantially depended on this timespan. Still, the majority of blood samples in our study were taken not more than 5 five months apart. Therefore, our results do not provide information with regard to larger timespans.

Assumed that false positive case classifications are rare, the biomarker-associated risk estimates from observational studies will be biased towards the null by imprecision in the measurements [26]. The exemplary correction of hypothetically observed risk estimates by the ICCs assessed in the present study demonstrated two insights. First, qualitative inference about the risks related to observed levels of circulating RBP4 and omentin-1 seems not to be severely biased by biological variation of these markers within individuals. Second, even for biomarkers with excellent reliability the underestimation of the “truly” associated risk induced by the random measurement error related to single time point biomarker measurements seems relevant in some regards. For a risk estimate of 1.5 correcting for the omentin-1-related ICC of 0.80 would induce a change in estimate over 10%, while correcting a risk estimate of 3.5 by the RBP4-related ICC of 0.77 would lead to a 45% higher estimated risk. In the future, in epidemiological studies, reporting ICC-corrected risk estimates in sensitivity analyses might be a convenient possibility to quantify the bias induced by imprecision of the measurement due to variation of RBP4 and omentin-1 within individuals.

Strengths of our study include the good characterization of participants that allowed us testing robustness of our results according to several strata and performing sensitivity analyses. Furthermore, regarding age and BMI distributions our population is likely to resemble conditions among prospective cohorts in which the relations of RBP4 and omentin-1 with the incidence of metabolic and cardiovascular diseases are likely to be investigated in future. As our population was predominantly from European descent, caution needs to be taken in generalizing our results to other ethnicities. Our study population represented a normal population and it is unclear in how far our results can be generalized to cohorts with diseased subjects. Of note, we most likely did not include participants with severe renal diseases and markers of kidney function were not available in this validation study. Glomerular filtration rate, however, is a main determinant of RBP4 levels, with impaired kidney function being related to unphysiologically high circulating concentrations [13, 27]. Importantly, we did not evaluate stability of RBP4 levels in patients with impaired renal function and our results might not be generalizable to study populations enriched with such patients. Generalization of our study results for use in error correction models might also be hampered by sampling and measurement procedures. We used EDTA plasma and the ELISAs were validated for the use of EDTA plasma. Care should be taken if other anticoagulants or other assays are used. Regarding RBP4, the quantification in ELISAs was generally criticized and new mass spectrometry-based approaches were proposed [28]. Such approaches are of interest as they provide additional information on the posttranslational modifications of RBP4. Still, ELISAs provide the most cost effective and widespread method to quantify RBP4 in the blood in large cohorts and our results encourage their use in such studies.

In conclusion, our results indicate that single measurements of RBP4 and omentin-1 provide highly reliable estimates for the concentration of these adipokines in individuals. Thus, it seems reasonable to rely for risk estimates on concentrations of RBP4 and omentin-1 from single baseline samples in observational studies.
